# *Rickettsia* spp. in Ticks, Poland

**DOI:** 10.3201/eid1503.080711

**Published:** 2009-03

**Authors:** Tomasz Chmielewski, Edyta Podsiadly, Grzegorz Karbowiak, Stanislawa Tylewska-Wierzbanowska

**Affiliations:** National Institute of Public Health, Warsaw, Poland (T. Chmielewski, E. Podsiadly, S. Tylewska-Wierzbanowska); W. Stefanski Institute of Parasitology of the Polish Academy of Sciences, Warsaw (G. Karbowiak)

**Keywords:** Rickettsiae, ticks, Poland, dispatch

## Abstract

Ticks are recognized as the main vectors and reservoirs of spotted fever group rickettsiae. We searched for the most prevalent *Rickettsia* spp. in Poland and found *R. slovaca* and *R. helvetica* bacteria in ticks in southern and central Poland; *R. raoulti* was found in ticks in all parts of Poland.

Ticks are ectoparasites infesting many mammals, including humans and their pets. In Poland, *Ixodes ricinus* ticks are widely distributed throughout the country whereas *Dermacentor reticulatus* ticks are limited to the northeast region. Both *I. ricinus* and *D. reticulatus* ticks are known to be the main vectors and reservoirs of the spotted fever group rickettsiae (SFG) worldwide. Detection of the SFG pathogens requires a 2-step procedure: PCR and sequencing of selected genes (16S rRNA, citrate synthase [*gltA*], outer membrane protein A [*ompA*], *ompB,* and 17-kDa protein) (*1*–*6*). The aim of the present study was to identify and characterize rickettsial species occurring in ticks in Poland.

## The Study

We collected 214 ticks in 3 regions of Poland: the Warsaw region (central Poland) (107 *I. ricinus* ticks), the Radomsko region (southern Poland) (47 *I. ricinus* ticks), and the Bialowieza Primeval Forest National Park (northeastern Poland) (60 *D. reticulatus* ticks). The specimens were collected from April 2005 through August 2007 and identified on the basis of morphologic characteristics. All *I. ricinus* ticks were obtained from dogs and cats, and *D. reticulatus* ticks were collected from vegetation.

DNA was extracted from *I. ricinus* specimens by using the QIAamp DNA Tissue Kit (QIAGEN, Hilden, Germany). *D. reticulatus* specimens were crushed in Eppendorf (Hamburg, Germany) tubes, after which DNA extraction was performed by boiling the specimens in 200 μL of 0.7 M NH_4_OH for 30 min.

Bacterial DNA was examined for the *Rickettsia* spp. *gltA* gene by using *Rp*CS.409d and *Rp*CS.1258n primers. Each positive specimen was amplified with paired primers *Rr*190-70 and *Rr*190-701, which were specific for the SFG rickettsiae *ompA* gene. In the absence of amplifiable fragments of the *ompA* gene, molecular identification was conducted by using PCR with paired primers *Rr*17.61p and *Rr*17.492n, which are able to anneal the specific 17-kDa outer membrane protein gene (Table 1).

**Table 1:** Oligonucleotide primers used for PCR amplification and sequencing of rickettsial species in ticks, Poland, 2005–2007

No.	Primers	Fragment gene (size, bp)	Nucleotide sequences (5′ → 3′)	Reference
1	*Rp*CS.409d	Citrate synthase (850)	CCTATGGCTATTATGCTTGC	(*5*)
	*Rp*CS.1258n		ATTGCAAAAAGTACAGTGAACA	
2	*Rr*190-70	Outer membrane protein A (632)	ATGGCGAATATTTCTCCAAAA	(*3*)
	*Rr*190-701		GTTCCGTTAATGGCAGCATCT	
3	*Rr*17.61p	17-kDa genus-common antigen (434)	GCTCTTGCA ACT TCT ATG TT	(*7*)
	*Rr*17.492n		CATTGTTCGTCAGGTTGGCG	

The QIAquick PCR Purification Kit (QIAGEN) was used to purify PCR products for sequencing. All amplicons were sequenced with the ABI Prism 377 DNA Sequencer (Applied Biosystems, Foster City, CA, USA) in accordance with the manufacturer’s recommendations. All sequences were edited by using the AutoAssembler software (Applied Biosystems), identified with the BLAST software (http://blast.ncbi.nlm.nih.gov/Blast.cgi), and compared with sequences available in GenBank.

The DNA of *Rickettsia* spp. was found in 70 (32.7%) *I. ricinus* and *D. reticulatus* ticks by using PCR and primers specific for the *gltA* gene. All 70 tick samples that tested positive for the genus *Rickettsia* were subjected to amplification with primers specific for the *ompA* gene. Twenty-eight *I. ricinus* and 34 *D. reticulatus* ticks were positive in PCR for the specific *ompA* fragment. In DNA samples extracted from 8 *I. ricinus* specimens (7 negative for the *ompA* gene and 1 having a faint reaction to the *gltA* gene), gene fragments characteristic of the 17-kDa protein were found.

Sequences of the *gltA* gene fragment (688 nt) from 62 samples were identical to the *R. raoultii* Marne and *R. raoultii* Khabarovsk strain sequences (GenBank accession nos. DQ365803.1 and DQ 365804.1). Results were confirmed by sequencing the *ompA* fragment; all 62 showed 99%–100% nucleotide similarity with the *ompA* gene (610 nt) of the *R. raoultii* Marne and the *R. raoultii* Khabarovsk strains (GenBank accession nos. DQ365799.1 and DQ365801.1).

Seven sequences of the *gltA* gene fragment amplicons were identical with the *R. helvetica* *gltA* gene (GenBank accession nos. U59723.1 and DQ131912.1). These samples also had 100% identical sequences of the 17-kDa protein gene characteristic of *R. helvetica* (GenBank accession nos. EF392726.1 and AJ427881.1).

The sequence of 1 amplicon with primers specific for the *ompA* gene showed 99% similarity with the *R. slovaca* *ompA* gene (GenBank accession no. U43808.1). Sequencing of PCR products with primers specific for the *gltA* and 17-kDa protein gene were not conducted due to the small amount of DNA in the sample and also due to poor amplification.

*R. raoultii* DNA was detected in 34 (56.7%) of the 60 *D. reticulatus* specimens from Bialowieza and in 28 (18.2%) of the 154 *I. ricinus* specimens tested, including 25 (23.4%) of 107 from the Warsaw region in central Poland and 3 (6.4%) of 47 *I. ricinus* specimens from the Radomsko region in the south. *R. helvetica* and *R. slovaca* were noted exclusively in DNA extracted from the *I. ricinus* ticks. In 3 (2.8%) of the 107 ticks from the Warsaw area, DNA of *R. helvetica* was found. In the Radomsko area, 4 (8.5%) of 47 specimens had *R. helvetica* DNA, and 1 (2.1%) had *R. slovaca* DNA (Table 2).

**Table 2:** *Rickettsia* spp. detected in ticks (N = 214) from 3 regions of Poland, 2005–2007

*Rickettsia* sp.	Total no. (%) ticks positive	Białowieża,* *Dermacentor reticulatus* ticks (n = 60), no. (%) positive	Radomsko,† *Ixodes ricinus* ticks (n = 47), no. (%) positive	Warszawa,‡ *I. ricinus* ticks (n = 107), no. (%) positive
*R. helvetica*	7 (3.3)	0	4 (8.5)	3 (2.8)
*R. slovaca*	1 (0.5)	0	1 (2.1)	0
*R. raoultii*	62 (29.0)	34 (56.7)	3 (6.4)	25 (23.4)

*Eastern Poland. †Southern Poland. ‡Central Poland.

## Conclusions

The DNA of *R. raoultii* was found in *Ixodes* spp. ticks in southern Poland and in *Dermacentor* spp. ticks in northeastern Poland. Until recently, *R. raoultii* had been reported only in *D. nutallii*, *Rhipicephalus* sp., and *Dermacentor* sp*.* ticks in Europe and Asia (i.e., Siberia and the Astrakhan area) (*8*–*11*).

SFG rickettsiae in Poland have been previously noted solely in *D. reticulatus* ticks (*12*). The *gltA* gene fragment sequence demonstrated similarities with *R. honei* and other unidentified SFG rickettsiae. The nucleotide sequences of amplified fragments of the *ompA* gene were 98% homologous to RpA4 *Rickettsia* sp.

Our findings show that *R. raoultii* occur in all regions of Poland. These bacteria were noted in 18.2% of *I. ricinus* and 56.7% of *D. reticulatus* specimens. Their occurrence in various species of ticks may suggest that they are capable of being distributed all over Europe.

Although the pathogenic role of these genotypes has not yet been established, their ability to cause infection cannot be ruled out. In fact, the RpA4 strain has been isolated from a patient with symptoms resembling those of *R. slovaca* infection (D. Raoult, unpub. data) (*10*). *R. slovaca*, a member of the SFG rickettsiae, was isolated from *D. marginatus* ticks in Slovakia in 1968 (*13*). Research has established that this bacterium is widely distributed and has been isolated from *D. marginatus*, *I. ricinus*, *Haemaphisalis* spp., and Argas ticks in many countries (*13*). From a medical standpoint, *R. slovaca* infection seems to be the most important SFG rickettsiosis in central Europe because of the severe and characteristic symptoms that occur after being bitten by these ectoparasites.

*R. helvetica*, which was detected in Switzerland in 1979, is also widespread in Europe. This species has recently been found in *I. ricinus* ticks in the northern part of Poland (*14*). Our study also confirms the occurrence of *R. helvetica* in central and southern Poland. These reports suggest that *R. helvetica* can infect *I. ricinus* ticks and may be extensively distributed in several European countries, including Poland. The pathogenicity of *R. helvetica* as a self-limiting illness associated with headache, myalgias, rash, or eschar has been confirmed. Also, several patients with perimyocarditis associated with *R. helvetica* have been observed in Sweden (*15*).

Our findings indicate that SFG rickettsiae transmitted by ticks could penetrate biotopes in various parts of Europe. Though the pathogenicity of the newly recognized species of the genus *Rickettsia* has not yet been proven definitively, it is prudent for clinicians in Poland and other European countries to be alert to possible appearances of infections caused by these pathogens.
